# The Influence of Low-Head Dams on the Biodiversity of Wintering Waterbirds in China’s Xin’an River Basin

**DOI:** 10.3390/biology15100757

**Published:** 2026-05-09

**Authors:** Fengming Dou, Xueyun Li, Chao Yu

**Affiliations:** 1 College of Animal Science and Technology, Jilin Agricultural Science and Technology College, Jilin 132101, China; 2 College of Life and Environmental Sciences, Huangshan University, Huangshan 245021, China; 3 Environment Conservation Research Centre of Xin’an River Basin, Huangshan University, Huangshan 245021, China; 4 School of Environmental Studies, China University of Geosciences, Wuhan 430074, China

**Keywords:** Xin’an River Basin in China, low-head dams, overwintering waterbirds, community diversity

## Abstract

The rivers in the middle and lower reaches of the Yangtze River are important wintering and migration stopovers for waterbirds. The hydrological characteristics of rivers directly affect the habitats of overwintering waterbirds and thus lead to changes in the diversity of overwintering waterbirds. The construction of artificial low-head dams has altered the natural hydrological processes of rivers, and therefore, investigating their influence on the composition of wintering waterbird communities is of great significance for the conservation and management of waterbirds. This study was carried out in the Xin’anjiang River Basin from October 2021 to March 2022, with 11 low-head dams selected as the research sites. Utilizing the sampling method, it investigated the species and abundance of wintering waterbirds in both the catchment and tailwater zones of these dams. Subsequently, the diversity of overwintering waterbirds in the two aforementioned zones was calculated, and their inter-zonal differences were analyzed and compared. The results of the study indicate that there are significant differences between the catchment area and the tailwater area of the “ZSJC” Dam (Z = 1.945, *p* = 0.001), whereas no significant disparities are observed in the species count and abundance of wintering waterbirds using that particular area between the catchment and tailwater areas of other dams. Compared with the catchment areas, the tailwater areas of the dams exhibit a more concentrated and abundant distribution of overwintering waterbirds, while the distribution of overwintering waterbirds in the catchment areas is more uniform than that in the tailwater areas. The 11 dams under study all demonstrated spatial turnover advantages, suggesting that catchment areas and tailwater areas make comparable contributions to β diversity. Bivariate correlation analysis in SPSS detected a significant correlation between dam vertical length and β diversity. In summary, low-head dam construction significantly affects the alpha diversity, beta diversity, abundance, and community composition of wintering waterbirds by modifying hydrological conditions and habitat structure in the Xin’an River Basin. This study provides a scientific basis for waterbird protection and low-head dam management.

## Simple Summary

Rivers in the middle and lower Yangtze River are key wintering and stopover sites for waterbirds. River hydrology shapes waterbird habitats and diversity, and artificial low-head dams have changed natural hydrological processes. Assessing their impacts on wintering waterbird communities is vital for conservation. From October 2021 to March 2022, we surveyed wintering waterbirds at 11 low-head dams in the Xin’anjiang River Basin, comparing species, abundance, and diversity between dam impoundment and tailwater zones. Only the Zhongsheng Jiancai (ZSJC) Dam showed significant differences between zones. Tailwater zones supported more concentrated waterbird distributions, while impoundment areas had more even distributions. All dams contributed similarly to β diversity, and dam vertical length was significantly correlated with β diversity. This study clarifies low-head dam impacts on waterbird communities, supporting waterbird protection and dam management.

## 1. Introduction

As a transitional zone between rivers and land, wetlands boast a superior natural environment and are rich in biological resources. The abiotic and biotic components within wetlands are interconnected and influence each other through material cycling, energy flow, and information transmission, forming a unique ecosystem. Peculiarly, wetlands constitute a critical component of natural ecosystems, serving functions such as supplying clean water to the global environment, facilitating adaptation to climate change (albeit occasionally linked to adverse climatic impacts), and safeguarding biodiversity [1]. Concurrently, wetlands play a pivotal role in landscape configuration and the functional operation of water resource and environmental systems, while providing substantial underpinnings for ecosystem services [2].

As a core habitat sustaining waterbird diversity, wetland ecosystems are of vital importance to overwintering waterbirds, providing them with essential resources for survival and reproduction. Simultaneously, waterbirds play integral roles in wetland ecosystems and maintain close linkages with these systems. They deliver a wide array of ecosystem services through diverse ecosystem functions and can serve as effective indicators for monitoring wetland health. The relationship between biodiversity and ecosystem functioning (BEF) has been a central topic in ecological research over the past two decades [3]. Nevertheless, the global community still confronts numerous challenges related to the loss of waterbird diversity, ecosystem degradation, and habitat conservation. Existing studies have demonstrated that the degradation of wetland-hydrological environment and the loss of wetland-habitat functions disrupt the biodiversity-ecosystem functioning relationship, thereby impairing habitat quality and waterbird diversity [4]. Furthermore, the conservation of waterbird diversity is closely associated with the normal functioning of wetland ecosystems, including nutrient cycling, energy storage, and primary productivity, especially the complex preferences and sensitivity of waterbirds to habitats, which also influence biological interactions.

In general, wetlands of different sizes have varying impacts on the abundance and diversity of waterbirds. Larger wetlands are usually characterized by abundant resources and structurally complex, well-established biodiversity. However, this is not universally applicable, as some large wetlands may be dystrophic and lack sufficient food resources for waterbirds. The increase in water surface area may provide sufficient food and environmental resources to meet the requirements of waterbirds. When food availability in their wetland habitats becomes inadequate due to external disturbances, waterbirds may shift their habitats and adjust their foraging behavior to fulfill nutritional demands and improve body condition [5]. Concurrently, waterbirds will also forage in the surrounding landscape to meet their energetic needs. These processes thus exert corresponding effects on the richness and diversity of overwintering waterbirds. Furthermore, habitat selection by waterbirds is closely linked to foraging behavior, which is strongly associated with the distribution and abundance of food resources in a habitat [6], including food quantity, resource richness, periodic fluctuations in food availability, and the vegetative water content. Each year in early winter, abundant water resources and food resources attract large numbers of waterbirds to inhabit the area. Waterbirds are often regarded as important indicators of the health status of aquatic ecosystems, and shifts in their community structure and diversity changes can intuitively reflect the quality of the ecological environment [7]. Collectively, these factors frequently modulate the environmental carrying capacity of wetlands for waterbirds.

Numerous previous studies have demonstrated that the habitat use and spatial distribution of waterbirds are influenced by a variety of factors: (1) Deep water levels within the basin may inundate wintering habitats such as shallow shoals and mudflats, while excessive flow velocity and increased turbidity in the lake water may prevent waterbirds from foraging on the water surface, thereby affecting their foraging activities and abundance of birds using that particular [8]. (2) Insufficient light in deep water areas hinders photosynthesis in aquatic plants, reducing the food supply for waterbirds and potentially leading to a decrease in invertebrates and fish, which in turn affects the number of waterbirds foraging [9]. (3) Shallow water areas have higher habitat heterogeneity and provide diverse food resources for waterbirds. The wide range of food types supports multiple feeding guilds with different foraging behaviors, which in turn leads to higher species diversity and greater abundance of wintering waterbirds in these habitats [10]. Shallow shoals and wetlands are valuable ecosystems for maintaining biodiversity. Their importance stems primarily from the greater variety of food resources they provide, which allows them to support higher abundances of wintering waterbirds and is thus crucial for their survival. (4) Human disturbance also has a negative impact on the distribution of waterbirds. Human activities and infrastructures keep waterbirds in a persistent state of alteration [11], reducing their foraging behavior and causing them to leave their habitats frequently, leading to a decrease in waterbird abundance.

**Figure 1:**
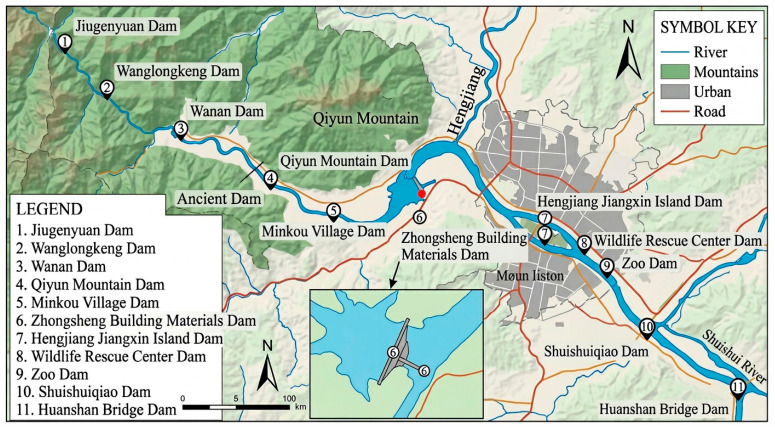
Schematic diagram of the research area, depicting the spatial arrangement of survey grids utilized in our research investigation.

The Xin’an River is located at the junction of Anhui Province and Zhejiang Province in China, stretching a total length of 373 km and draining a basin area of more than 11,000 km^2^. The source of the Xin’an River lies in Xiuning County, Huangshan City, from which the river flows eastward into western Zhejiang, passes through Chun’an and Jiande, and eventually empties into the Lanjiang River, forming a segment of the Qiantang River. The Xin’an River Basin is geographically situated in the northern subtropical region with a warm climate. However, the construction of dams in the Xin’an River Basin in recent years, especially a large number of low-head dams (low-head water retaining dams), has disrupted river continuity and altered hydrological dynamics, causing changes in the relevant water environment and thus affecting the survival and reproduction of organisms [12]. After the reservoir is filled with water, the water level rises and submerges the vegetation, resulting in a decrease in vegetation available for forage and an expansion of the aquatic environment, causing changes in the area of the watershed and dam tail that can accommodate water birds. More directly, parameters such as the height, length, and width of dams determine the depth and water surface area of the catchment area (The static water habitat formed after the dam blocked the water flow), as well as the hydrological state of the tailwater area. Meanwhile, hydropower dams disrupt the downstream runoff regimes, which in turn affects the vegetation distribution in river shoals and ultimately impacts the diversity of wintering bird communities [13]. In addition, the construction of dams will increase the sediment capacity of the reservoir area, leading to sedimentation and slower flow velocity, resulting in an increase in the density and biomass of planktonic animals, which indirectly affects the food structure obtained by waterbirds. Consequently, the establishment of dams to some extent changes the habitat quality, availability of food resources, and activity patterns of waterbirds. It is worth mentioning that the construction of surrounding ecological parks or scenic spots has increased pedestrian flow, which has a certain impact on the ecological environment of rivers, exacerbating anthropogenic disturbance to waterbirds (Figure 1).

Accordingly, this research systematically investigates the impacts of low-head dams on overwintering waterbird diversity and richness in the Xin’an River Basin, thereby facilitating an exploration of the ecological relationships between overwintering waterbirds and dam physical factors, providing a scientific basis for watershed ecosystem conservation and management, which is of great significance for maintaining the biodiversity and ecological balance of the Xin’an River Basin.

## 2. Materials and Methods

### 2.1. Overview of Xin’an River Basin

The Xin’an River Basin is located in the central eastern region of China and the transitional zone between the north and south climates, spanning two provinces, Anhui and Zhejiang. Its geographical coordinates range from 117°38′–119°21′ E longitude to 29°11′–30°20′ N. The watershed includes 10 districts and counties, with a total area of 11,190 km^2^, including 4715 km^2^ in Zhejiang Province and 6475 km^2^ in Anhui Province. The main tributaries of the Xin’an River system include the Hengjiang River, Lianjiang River, Changxi River, Jieyuan River, Dushui River, and Zhanghe River. The upstream is mountainous canyons with pebble beds and fast flow velocities. The middle reaches are the Kuangu Basin, with the Shexian Plain along the coast. The downstream is a reservoir area, with winding lake shores and dense islands. The average annual precipitation in the basin is about 1760 mm, with an average annual runoff of about 10 billion cubic meters. The seasonal differences in water volume are significant, with summer being the flood season and winter being the dry season. The annual average temperature is 17 °C.

### 2.2. Investigation Methods

From mid-October 2021 to late March 2022, during the entire wintering period, the study area was the rivers in the Huangshan region (including the Shuoshui tributary, the Hengjiang tributary, and the Nan’anjiang main stream). The bird population was surveyed using the sampling point method, with the location fixed at the edge of the wetland. The survey time was 4 h after sunrise and 4 h before sunset. At each fixed survey point, three sub-sampling points were set, with each sub-sampling point spaced 100 m apart. Each sub-sampling point was surveyed for 5 min. The bird species and their numbers observed within these 5 min, as well as the bird species and their numbers determined based on the bird calls heard, were recorded using a single-lens reflex telescope (DiaScope 85T*FLD, 20–75×, ZEISS, Oberkochen, Germany) and a binocular telescope (CONQUEST HD, 10×, ZEISS). Finally, the data from the three sub-sampling points were aggregated to obtain the species and numbers of birds at the survey site, and then the changes in the bird community structure of the Nan’anjiang River Basin were analyzed. Each survey was conducted by at least two people, with one person responsible for observing the bird species and numbers, and the other person responsible for recording the observed data. River section division: The survey sites above the Lishui Bridge Dam and the Mei Lin Wetland were classified as the upper reaches of the Nan’anjiang River, the sites from the Huangdun Second Bridge to below the Yan Village were classified as the middle reaches, and the sites below the Yan Village were classified as the lower reaches of the Nan’anjiang River.

### 2.3. Data Analysis

All data were organized and standardized prior to analysis. Variables analyzed included: species richness, individual abundance, alpha diversity (Shannon–Wiener index, Pielou’s evenness index), beta diversity (total beta, turnover component Beta_sim, nestedness component Beta_sne), and dam structural parameters (vertical length, height, width). Statistical analyses were performed using IBM SPSS Statistics 26.0. Nonparametric Mann–Whitney U tests were used to examine differences in waterbird community metrics between impoundment and tailwater zones. Bivariate Pearson correlation analysis was used to explore relationships between dam morphological parameters and waterbird beta diversity. A significance level of α = 0.05 was applied for all statistical tests. Alpha and beta diversity indices were calculated to characterize community structure. Although the Shannon–Wiener index integrates richness and evenness, Pielou’s evenness was used independently to clarify whether community variation was driven by species richness or abundance distribution. Beta diversity partitioning was applied to distinguish between spatial turnover and nestedness components of community differentiation.

## 3. Results

### 3.1. Species Structure and Distribution Characteristics of Overwintering Waterbirds in the Xin’an River Basin

**Figure 2:**
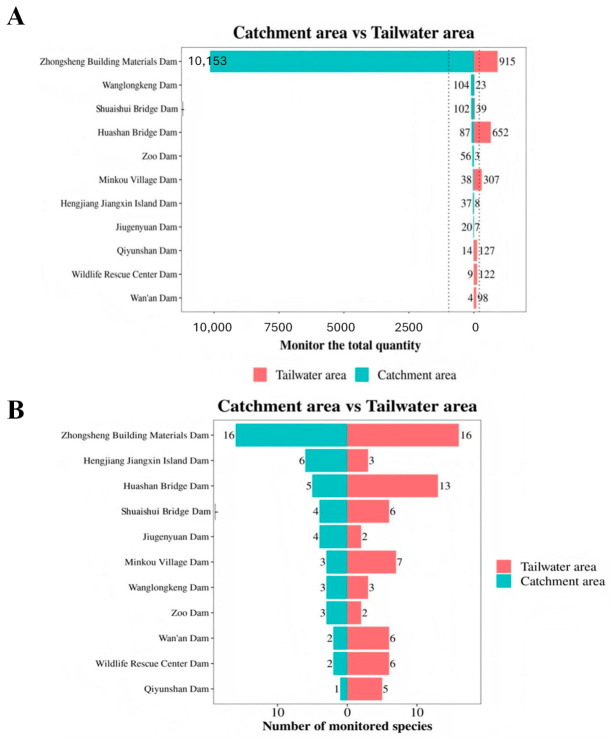
Number of registered species (**A**) and number of individuals (**B**).

According to the survey results (Table 1; Figure 2A,B), a total of 10,624 wintering waterbirds belonging to 49 species were recorded in the catchment areas of the 11 dams, with an average of 965.818 individuals per dam. The Kruskal–Wallis H test showed significant differences in the distribution of waterbird species across all sampled low-head dams (*p* < 0.05), indicating distinct habitat conditions among these dams in the Xin’an River Basin. Among them, the catchment area of the “Zhongsheng Building Materials” (ZSBM) dam yielded 16 species and 10,153 individuals of wintering waterbirds, representing the highest species richness and abundance, which reflects high biodiversity and favorable habitat conditions. In contrast, only 2 species and 4 individuals were found in the catchment area of the Wan’an (WA) Dam, and 1 species and 14 individuals at the “Qiyunshan” (Qys) Dam, both showing the lowest species richness and abundance of waterbirds. This may be attributed to limited food resources or unsuitable ecological conditions such as water level and vegetation for most waterbird species in the region. In addition, a total of 2301 wintering waterbirds from 69 species were monitored in the tailwater areas of all dams across the basin, with an average of 209.182 individuals per dam, suggesting substantial variation in ecological carrying capacity among different dam sections even within the same watershed.

Furthermore, 16 species and 915 individuals of wintering waterbirds were recorded in the tailwater area of the ZSBM Dam. The ZSBM Dam supported the highest number of waterbird species in both the catchment and tailwater zones, indicating rich avian resources and its status as the most important area for biodiversity conservation in the entire Xin’an River Basin. The tailwater area of the Zoo Dam contained only 2 species and 3 individuals, representing the lowest abundance among all tailwater sections.

The 11 studied low-head dams varied considerably in construction year, ranging from 1957 to 2015 (Table 2). This wide range in dam age indicates substantial differences in habitat development time, which may further contribute to the observed variation in waterbird assemblages among sites.

### 3.2. Analysis of Diversity Differences in Overwintering Waterbirds at Different Locations

**Figure 3:**
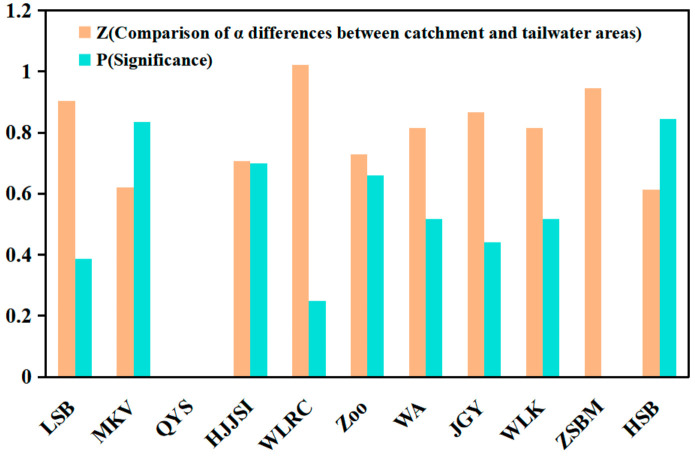
Pairwise differences in overwintering waterbird abundance between different locations, shown as *p*-values from Mann–Whitney U tests. Darker blue indicates lower *p*-values (greater differences).

Although water depth was not directly measured in this study, the impoundment zones upstream of dams were characterized by deeper, still water, whereas tailwater zones downstream were characterized by shallower water and slower currents. These relative differences in depth and hydrological conditions corresponded closely with the observed patterns of waterbird aggregation. Comparing the differences in alpha diversity of overwintering waterbirds between the catchment area and the dam tail water area (Figure 3), it was found that Z (“ZSBM” dam catchment area and dam tail water area) = 1.945, *p* = 0.001, had significant differences, while there were no significant differences in the number of overwintering waterbird species and abundances between other dam catchment areas and dam tail water areas; Z (the catchment area and tailwater area of the “LSB” dam) = 0.904, *p* = 0.388; Z (“MKV” dam catchment area and dam tail water area) = 0.621, *p* = 0.835; Z (“HJJXI” dam catchment area and dam tail water area) = 0.707, *p* = 0.699; Z (“WRC” dam catchment area and dam tail water area) = 1.021, *p* = 0.249; Z (the watershed and tailwater area of the “Zoo” dam) = 0.730, *p* = 0.660; Z (“WA” Dam catchment area and dam tail water area) = 0.816, *p* = 0.518; Z (the catchment area and tailwater area of the “JGY” dam) = 0.866, *p* = 0.441; Z (“WLK” Dam catchment area and dam tail water area) = 0.816, *p* = 0.518; Z (“HSB” dam catchment area and dam tail water area) = 0.614, *p* = 0.845. The “QYM” Dam cannot be calculated due to insufficient data.

**Figure 4:**
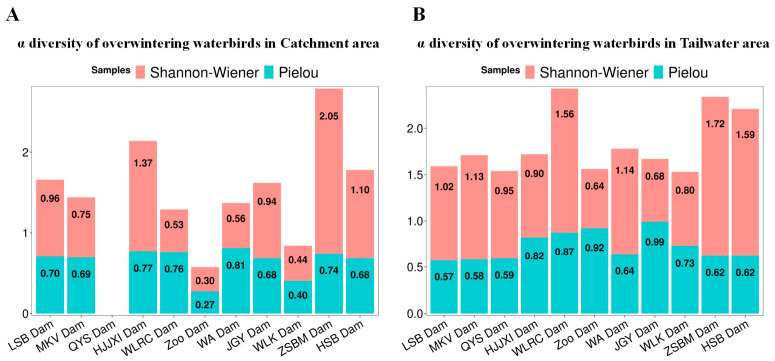
α diversity of overwintering waterbirds in catchment area (**A**) and tailwater area (**B**).

In the analysis of the alpha diversity index (Table 3, Figure 4A,B), based on Mann–Whitney U tests, the Shannon–Wiener diversity index was generally higher in the impoundment area than in the tailwater area, except for the “HJJXI” dam (1.37), “JGY” dam (0.94), and “ZSBM” dam (2.05). The Pielou evenness index shows that the catchment area is larger than the tailwater area in the “LSB” dam, “MKV” dam, “WA” dam, “ZSBM” dam, and “HSB” dam, while the catchment area is smaller than the tailwater area in the “QYS” dam, “HJJXI” dam, “WLRC” dam, “Zoo” dam, “JGY” dam, and “WLK” dam. Overall, in terms of diversity, the water birds overwintering in the dam tail area are more concentrated and abundant compared to the catchment area, with the species in the “ZSBM” dam tail area being the most diverse. In terms of uniformity, the distribution of overwintering waterbirds in the catchment area is more uniform compared to the tailwater area, with the “JGY” dam tailwater area having the most uniform distribution.

**Figure 5:**
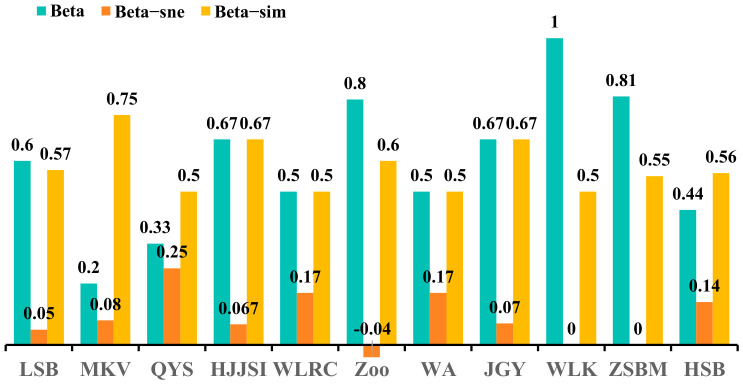
Pairwise β-diversity of wintering waterbird communities among sampling sites in the Xin’an River Basin.

In the analysis of the β diversity index (Figure 5), the β diversity of the overall catchment area and dam tail water area is Beta = 0.789, Betanes = 0.028, and BetaSim = 0.531. Among all the catchment and tailwater areas, the β diversity of waterbirds in the “MKV” dam is the smallest (Beta = 0.200, Betasne = 0.083, BetaSim = 0.750), indicating that the similarity of overwintering waterbirds in the “MKV” dam tailwater area and catchment area is the highest. The “WLK” dam has the highest β diversity in the catchment and tailwater areas (Beta = 1.000, Betanes = 0.000, BetaSim = 0.500). These research data all indicate that the 11 Xin’anjiang River basins studied have an advantage in spatial turnover, with similar contributions to β diversity from the catchment area and dam tail water area. This further suggests that species composition has undergone significant changes between upstream and downstream, and this change may be mainly driven by the natural environment rather than random diffusion. This result also highlights the role of the low-head dam as an ecosystem filter in restructuring community structure to a certain extent.

### 3.3. Analysis of the Relationship Between Bird Diversity and the Structural Characteristics of Lower Head Dams

In order to reveal the correlation between various parameters of dams (dam height, actual length, width, and vertical length) and the β diversity of waterbirds, the bivariate correlation analysis function in SPSS was used to analyze it. The results showed (Table 4 and Table 5) that there was a significant correlation between the vertical length of dams and β diversity (r = 0.710, *p* = 0.021), while there was no significant correlation between dam height and β diversity (r = 0.158, *p* = 0.800).

## 4. Discussion

The spatiotemporal patterns of waterbird community diversity and similarity form the theoretical basis for understanding the environmental adaptation mechanisms of birds. The overall changes in waterbird diversity observed in this study were mainly driven by five key factors: hydrological conditions (water depth and flow), habitat heterogeneity and water surface area, food availability, anthropogenic disturbance, and dam structural characteristics. Diversity indices are determined by species richness and evenness, which reflect spatiotemporal variation in communities [14]. Higher richness and evenness generally correspond to higher diversity. Our results indicated that the Shannon–Wiener diversity index was higher in tailwater zones than in impoundment zones for most dams, except HJJXI, JGY, and ZSBM dams. No significant difference in Pielou evenness was detected among dams (*p* > 0.05). This pattern was closely related to habitat characteristics: tailwater zones had slower flow, stable water levels, and shallow habitats that supported richer foraging resources and more bird species. By contrast, impoundment zones were deeper with relatively homogeneous habitats, leading to lower species diversity. The non-significant variation in evenness may be due to dominant species in both habitat types. These results are consistent with studies reporting that higher habitat heterogeneity promotes species coexistence and diversity [15,16].

In addition, although actual water depth was not quantitatively measured in this study, the impoundment zones upstream of dams were characterized by relatively deeper, static water bodies, whereas tailwater zones were characterized by shallower water and slower flow velocities. These relative differences in water depth and hydrological conditions may be key drivers underlying the observed patterns of waterbird aggregation and community structure across the study area. Notably, only the Zhongsheng Building Materials (ZSBM) Dam showed a statistically significant difference in waterbird community structure between the impoundment and tailwater zones. This unique pattern may be attributed to three key factors: water level conditions, habitat surface area, and human disturbance intensity. First, the ZSBM Dam has a clearer hydrological contrast, with deeper and more stable impoundment water levels versus shallower, slower-flowing tailwater habitats that provide more favorable foraging resources. Second, the dam exhibits a larger water surface area and higher habitat heterogeneity than other dams, supporting more distinct community assemblages between the two zones. Third, this dam is located in a relatively natural area with lower anthropogenic disturbance, which preserves strong habitat differences between upstream and downstream sections. In contrast, other dams experience higher human interference or more homogeneous hydrological conditions, resulting in non-significant between-zone differences.

Specifically, β diversity is a crucial metric in ecology that quantifies the variation in species composition among different sites. It aids in elucidating the mechanisms underlying the assembly of species communities across spatial dimensions [17], while also encompassing the differences in species composition among communities in distinct habitats and the rate of species turnover along environmental gradients. As the number of shared species among different communities or along specific environmental gradients decreases, the similarity between communities correspondingly declines, leading to an increase in β diversity. The results of this study indicate that, when accounting for variations in the vertical dimensions of dams, significant changes occur in the waterbird diversity between the reservoir and tailwater areas of dams within the Xin’an River Basin, resulting in differential β diversity values. Moreover, within the research framework of wintering waterbird communities established in this study, the spatial distribution pattern of β diversity not only reflects the compositional structure of bird communities in different zones of low-head dams across spatial dimensions, but also reveals the mechanisms sustaining the structure and diversity of wintering bird communities within the Xin’an River Basin.

Furthermore, animals exhibit selective utilization and adaptation to their habitats. For instance, tailwater areas typically offer greater food resources for waterbirds to meet their energy demands during the wintering period. Additionally, both sides of the dams are rich in fish and shrimp resources, providing ample foraging opportunities for waterbirds. Shallow waters, muddy areas, and relatively eutrophic zones offer diverse habitats for a wider range of waterbird species, thereby significantly enhancing waterbird diversity. Shallow-water environments also increase the likelihood of successful foraging, enabling waterbirds to adapt flexibly between biodiverse shallow areas and less biologically productive zones. Waterbirds demonstrate rapid aggregation responses to such conditions, making them ideal indicator species for ecological changes [18]. The results of this study further indicate that tailwater areas of low-head dams, such as the “ZSBM” Dam, “LSB” Dam, “MKV” Dam, “QYS” Dam and “WLK” Dam, host relatively rich species diversity, consistent with findings from previous studies. Notably, areas surrounding dams such as the “HJJXI” Dam, “JGY” Dam and the “Zoo” Dam are characterized by high levels of human activity, including tourism and infrastructure development. Intense anthropogenic disturbance not only directly forces waterbirds to leave but may also indirectly affect their breeding behaviors and energy accumulation. For wintering waterbirds in particular, a stable environment is crucial for surviving the cold season. In contrast, regions such as the “QYS” Dam, “WA” Dam, and “WLK” Dam are surrounded by natural landscapes like woodlands and farmlands, where human activity is relatively minimal. These areas provide more stable habitats for waterbirds, resulting in longer residence times and more consistent observations of both individual numbers and species diversity. This suggests that the level of anthropogenic disturbance is another key factor influencing waterbirds’ selection of low-head dam areas as habitats. Therefore, based on the findings of this study, the management and conservation of low-head dams in the Xin’an River Basin should carefully consider the intensity of anthropogenic disturbance around different dams. Priority should be given to protecting areas with lower disturbance levels and higher habitat heterogeneity, while mitigating the negative impacts of human activities on waterbird habitats through rational planning. Such measures will help create more suitable living conditions for wintering waterbirds. Furthermore, we strengthened the data mining work to analyze the relationships between diversity indices and key environmental parameters. The results showed that water surface area, water level, and geomorphological and hydrological features of the tailwater area were significantly correlated with both α diversity and β diversity of wintering waterbirds. Larger water surface area and stable water level in the tailwater area could provide more suitable foraging and roosting habitats, thus increasing the diversity indices. The geomorphological complexity and hydrological stability of the tailwater zone were also key factors affecting the spatial distribution and community differentiation of waterbirds. In addition, dam age represents another important factor that may influence waterbird communities. Older dams have had sufficient time for habitat succession, leading to more stable hydrological conditions, well-developed aquatic vegetation, and richer food resources such as invertebrates and fish. In contrast, newer dams are still in an early stage of ecosystem development, with simpler habitats and lower food availability. The wide age range of dams in this study (from several years to several decades) may partially explain the strong differences in species richness, abundance, and diversity observed across the 11 sites. Therefore, dam age acts as an important indirect driver of habitat availability and waterbird assemblage structure in the Xin’an River Basin.

Correspondingly, the composition and quantity of waterbird communities are often influenced by both biotic and abiotic factors. The spatial distribution of the abundance composition and quantity of wintering waterbirds is closely related to the physical parameters of dams. The height, length and width of dams can affect the water level of rivers and the natural regulation of rainfall, leading to changes in the hydrological environment near low-head dams and subsequently influencing the community structure of birds. The results of this study also confirm this assertion. The data analysis of this study shows that the vertical height of the dam has a positive correlation with the impact on the waterbird community and its β diversity, but the correlation between height and β diversity is not significant. In addition, as the vertical length of the dam increases, β diversity also increases, resulting in greater differences in the waterbird communities between the catchment area and the tailwater area. The increase in vertical height of dams leads to a corresponding rise in the impoundment water level, while the area, perimeter, and shape of rivers and lakes influence the local heterogeneity of bird community species composition, and the increase in aquatic environment area also provides more foraging resources for waterbirds [19]. At the same time, the height of the dam also hinders the passage of fish in the river, reducing the opportunities for fish and shrimp and crabs to spawn, forage and reproduce in the river, making it difficult for them to swim upstream and causing them to gather in large numbers in the river channel, increasing the predation rate of waterbirds. These factors also affect the community structure of wintering waterbirds to a certain extent.

## 5. Conclusions

In summary, this study systematically analyzed the α and β diversity of overwintering waterbirds in the impoundment and tailwater areas of 11 low-head dams in the Xin’an River Basin. The results revealed notable differences in both α and β diversity between the two areas, with spatial turnover being the dominant component of β diversity. These results are consistent with similar studies demonstrating that low-head dams create contrasting habitats that strongly affect waterbird diversity and community composition. The significant correlation between dam structural parameters and waterbird assemblages also aligns with findings from other regulated river systems. This study provides a scientific basis for waterbird conservation and low-head dam management in the Xin’an River Basin. By placing our results in the context of existing literature, we strengthen the generality of our findings and support the maintenance of regional biodiversity and ecological balance.

## Figures and Tables

**Table 1:** Statistics of overwintering waterbird species and quantities in different Low-Head dams.

Dam		Number of Registered Species	Number of Individuals
Catchment area:	Lvshui Bridge Dam (LSB)	4	102
	Minkou Village Dam (MKV)	3	38
	Qiyunshan Dam (QYS)	1	14
	Hengjiang Jiangxin Island Dam (HJJXI)	6	37
	Wildlife Rescue Center Dam (WLRC)	2	9
	Zoo Dam	3	56
	Wan’an Dam (WA)	2	4
	Jiugenyuan Dam (JGY)	4	20
	Wanglongkeng Dam (WLK)	3	104
	Zhongsheng Building Materials Dam (ZSBM)	16	10,153
	Huashan Bridge Dam (HSB)	5	87
Tailwater area:	Lvshui Bridge Dam (LSB)	6	39
	Minkou Village Dam (MKV)	7	307
	Qiyunshan Dam (QYS)	5	127
	Hengjiang Jiangxin Island Dam (HJJX)	3	8
	Wildlife Rescue Center Dam (WLRC)	6	122
	Zoo Dam	2	3
	Wan’an Dam (WA)	6	98
	Jiugenyuan Dam (JGY)	2	7
	Wanglongkeng Dam (WLK)	3	23
	Zhongsheng Building Materials Dam (ZSBM)	16	915
	Huashan Bridge Dam (HSB)	13	652

**Table 2:** The species and numbers of wintering waterfowl at different dam sites and their construction times.

Dams	Number of Recorded Species	Total Individual Abundance	Construction Year
Lvshui Bridge Dam (LSB)	4	102	2011
Minkou Village Dam (MKV)	3	38	2008
Qiyunshan Dam (QYS)	1	14	1962
Hengjiang Jiangxin Island Dam (HJJXI)	6	37	2009
Wildlife Rescue Center Dam (WLRC)	2	9	2015
Zoo Dam	3	56	2013
Wan’an Dam (WA)	2	4	1957
Jiugenyuan Dam (JGY)	4	20	2006
Wanglongkeng Dam (WLK)	3	104	1999
Zhongsheng Building Materials Dam (ZSBM)	16	10,153	1990
Huashan Bridge Dam (HSB)	5	87	2011

**Table 3:** α diversity of overwintering waterbirds in different locations.

Dam		Shannon–Wiener	Pielou
Catchment area:	LSB Dam	0.96	0.70
	MKV Dam	0.75	0.69
	QYS Dam	-	-
	HJJXI Dam	1.37	0.77
	WLRC Dam	0.53	0.76
	Zoo Dam	0.30	0.27
	WA Dam	0.56	0.81
	JGY Dam	0.94	0.68
	WLK Dam	0.44	0.40
	ZSBM Dam	2.05	0.74
	HSB Dam	1.10	0.68
Tailwater area:	LSB Dam	1.02	0.57
	MKV Dam	1.13	0.58
	QYS Dam	0.95	0.59
	HJJXI Dam	0.90	0.82
	WRC Dam	1.56	0.87
	Zoo Dam	0.64	0.92
	WA Dam	1.14	0.64
	JGY Dam	0.68	0.99
	WLKDam	0.80	0.73
	ZSBM Dam	1.72	0.62
	HSB Dam	1.59	0.62

**Table 4:** Summary of dam characteristics and waterbird β diversity values across the study sites.

**Vertical Length of Dams and β Value**		**Vertical Length of Dams**	**β Value**
Vertical length of dams	Pearson Correlation	1	0.710 *
	Sig. (2-tailed)		0.021
	N	10	10
β value	Pearson Correlation	0.710 *	1
	Sig. (2-tailed)	0.021	
	N	10	10
**Dam Height and β Value**		**Dam Height**	**β Value**
Dam height	Pearson Correlation	1	0.16
	Sig. (2-tailed)		0.80
	Sum of square and cross product	12.07	0.21
	covariance	3.02	0.053
	N		5
β value	Pearson Correlation	0.16	1
	Sig. (2-tailed)	0.80	
	Sum of square and cross product	0.21	0.15
	covariance	0.05	0.037
	N	5	5

* Correlation is significant at the 0.05 level (2-tailed).

**Table 5:** The correlation between the vertical length/height of dams and the β diversity of waterbirds.

Dams	Dam Parameters and Waterbirds β Diversity Values			
	Vertical Length of Dams (m)	β Value	Dam Height (m) ^※^	β Value
QYS Dam	115	0.34	2.9	0.34
HJJXI Dam	100	0.34	1.59	0.34
WLRC Dam	148	0.5	-	-
Zoo Dam	186	0.8	3	0.8
WA Dam	194	0.5	6	0.5
ZSBM Dam	206	0.81	-	-
LSB Dam	217	0.6	-	-
MKV Dam	139	0.2	-	-
HSB Dam	288	0.44	4.8	0.44
WLK Dam	393	1	-	-
JGY Dam	85	0.32	-	-

※ The height parameters of the remaining six low-head dams have not been made public due to protection-related considerations and thus could not be incorporated into the statistical analysis.

## Data Availability

The original contributions presented in this study are included in the article. Further inquiries can be directed to the corresponding author.
